# Supplementary material for: Aqueous pKa prediction for tautomerizable compounds using equilibrium bond lengths
Source: Commun Chem. 2020 Feb 12;3:21. doi: 10.1038/s42004-020-0264-7 (PMC9814527; doi:10.1038/s42004-020-0264-7)
Supplement: Supplementary file 1 — Supplementary Information [file 42004_2020_264_MOESM1_ESM.pdf]

Supplementary Information

Aqueous pK$_a$ Prediction for Tautomerisable Compounds Using Equilibrium Bond Lengths

Beth A. Caine[a,b], Maddalena Bronzato[c], Torquil Frazer[c], Nathan Kidley[c], Christophe Dardonville[d] and Paul L. A. Popelier[a,b]

[a]School of Chemistry, University of Manchester, Great Britain,

[b]Manchester Institute of Biotechnology (MIB), 131 Princess Street, Great Britain,

[c]Syngenta AG, Jealott's Hill, Warfield, Bracknell, RG42 6E7, Great Britain,

[d]Instituto de Química Médica, IQM–CSIC, C/ Juan de la Cierva 3, 28006 Madrid, Spain

Contents

Supplementary Methods. 2-4

Supplementary Table 1. 5-12

Supplementary Note 1. 12

Supplementary Tables 2 - 6 13-18

Supplementary Note 2. 19-20

Supplementary Figure 1. 21

Supplementary Tables 7-13. 22-29

Supplementary Figures 2-3. 30-31

Supplementary References. 32

Supplementary Methods

Regression Approaches

Gaussian Process Regression (GPR)

We built our GPR model using the Python library called George. A gaussian process is a non-parametric model that defines a distribution over functions, which is updated according to the training data. The process is fully defined by two priors: the mean function (set to zero) and a covariance function $k(x, x')$ (also called the kernel, which in our case is a radial basis function or SE-ARD),

$$SE - ARD(x, x') = \exp\left(-\frac{1}{2} \sum_{d=1}^{N} \frac{|x - x'|^2}{\ell^2} \right) \quad (1)$$

The hyperparameters (l) for this kernel were found by maximising the log-likelihood function using the gradient descent BFGS algorithm (implemented by scipy), on the negative gradient of the log-likelihood function (therefore finding the maximum of the function). As there can be many local maxima, the optimiser was restarted with random weights 100 times in an attempt to find the global maximum.

Support Vector Regression (SVR)

We built our SVR model using the Python library scikit-learn. For a linear function, given a set of data $\{(x_1, y_1), \dots, (x_n, y_n)\}$ the goal of SVR is to find a function $f(x)$, i.e.,

$$f(x) = \langle w, x \rangle + b \quad (2)$$

where $\langle w, x \rangle$ corresponds to the dot product, and that has at most ε deviation from all training set y_i values, and is as flat as possible. In order to obtain a function that is as flat as possible the norm of w is minimised, which is a convex optimisation problem, i.e., the problem becomes,

$$\text{minimise} \quad \frac{1}{2}\|w\|^2 + C\sum_{i=1}^{n}(\xi_i - \xi_i^*) \quad (3)$$

$$\text{such that} \quad \langle w, x \rangle + b - y_i \le \varepsilon + \xi_i \quad (4)$$

$$y_i - \langle w, x \rangle + b \le \varepsilon + \xi_i^* \quad (5)$$

The loss function is the default in sklearn, which corresponds to the “epsilon-insensitive loss” (the L1 loss). In eq. 3 we have also introduced ξ_i and ξ_i^* as slack variables, which measure the deviation of training samples outside the ε-insensitive zone. These slack variables are used in ε-SVR as it can be a difficult task to find a function f that approximates all (x_n, y_n) with less than ε deviation for all points. C is the box constraint, which affects the trade-off between the model flatness and the extent that deviations larger

than epsilon are allowed in optimisation. C and ε were found via a GridSearch in scikit-learn using 7-fold cross validation RMSEE, along with ε.

Partial Least Squares (PLS)

We built our PLS models using the Python library scikit-learn. PLS was chosen over Multiple Linear Regression due to the possibility of multicollinearity between bond length features. PLS is a way of building a regression model using latent variable (LV) decomposition. The two matrices X and Y, containing the descriptors x and the target values y of the training set can be decomposed into a sum of f latent variables,

$$X = TP^T + E = t_f p_f' + E \quad (6)$$

$$Y = UQ^T + F = u_f q_f' + F \quad (7)$$

where T and U are score matrices, P and Q are loading matrices, and E and F are residual matrices, all for X and Y. For a latent variable,

$$u_f = b_f t_f \quad (8)$$

where b_f is the coefficient for the latent variable f. To identify an optimal number of LVs for each feature combination, models were constructed using 1 to *ndim* LVs (where *ndim* is the number of dimensions/features), and the model that returned the best RMSEE of the training set was selected.

Random Forest Regression (RFR)

We built our RFR model using the Python library called scikit-learn. Random Forest Regression is an ensemble method to regression. This means that multiple decision trees are used to arrive at a final output value, which is the average of the individual tree predictions. This is achieved by bagging, i.e. randomly sampling from the full set (by replacement) and a decision tree being trained on each training data sample, $\{(x_1, y_1), \dots, (x_n, y_n)\}$, where x_i, (i = 1, …, n), is a vector of descriptors and y_i is the target value (pK_a). For each tree the best split for each node is picked from a randomly selected subset of descriptors. The tree is then grown until further splits are not possible, and not pruned back. This procedure is repeated until enough trees have been grown. The number of estimators (n_{est}) and maximum depth were found in each case by applying a grid search (GridSearchCV in scikit-learn). The final hyperparameter values were chosen to minimize a 7-fold cross validation RMSEE.

Validation Metrics.

The r^2 score that we calculate to assess model predictability is produced via k-fold cross validation, where k=7. Hence, a 7th of the dataset is removed, and the remaining $6/7^{th}$ of the input features and observables are used to form the predictive equation. Predictions are then made for the 7th that was removed. The second 7th is then removed, and the first 7th joins the remaining $5/7^{th}$ to make up a new $6/7^{th}$ training set. When all 7 cycles are complete and all compounds have been predicted once, the following equation is used to obtain the r^2 value:

$$r^2 = 1 - \frac{\sum_i^N (y_{i,obs} - y_{i,pred})^2}{\sum_i^N (y_{i,obs} - \bar{y})^2} \qquad (9)$$

where $y_{i,obs}$ and $y_{i,pred}$ correspond to the observed and predicted values for each of the training set compounds, and $\bar{y}$ is the mean value of the observed values for the training set. The RMSEE values that we quote are derived from the following equation:

$$RMSEE = \sqrt{\frac{\sum_i^N (y_{i,obs} - y_{i,pred})^2}{N}} \qquad (10)$$

where $y_{i,obs}$ and $y_{i,pred}$ are defined as above and N is the number of compounds of the training set.The mean absolute error is defined as:

$$MAE = \frac{\sum_i^{N_{ext}} |y_{i,obs} - y_{i,pred}|}{N_{ext}} \qquad (11)$$

where $y_{i,obs} - y_{i,pred}$ is the residual error, and where i is now the test set compound and N_{ext} denotes the number of compounds in the external test set. These 7-fold CV metrics were calculated using cross_validate in sklearn in python for each of the 31 combinations of the 5 input features O-H, C-O, C=C, C-C and C=O: (5 x 1 bond length models) + (10 x 2 bond length models) + (10 x 3 bond length models) + (5 x 4 bond length models) + (1 x 5 bond length model) = 31.

External validation was also performed by calculation of the Mean Absolute Error (MAE) and by employing Roy's MAE evaluation criteria[11]. According to Roy, the two criteria that must be met by a "good" model are that:

1) the MAE must be less than 10% of the training set range, and

2) the MAE+3σ must be less than 20% of the training set range.

Here σ denotes the standard deviation of the absolute errors. If the model does not fit the above criteria then it can be deemed "moderate", that is, if for the second criterion 25% is used in place of 20%, or "poor" if it does not obey either criterion. The Root-Mean-Squared-Error of Prediction (RMSEP) calculated for the test set is also used to evaluate model prediction accuracy.

Supplementary Table 1. Structures, pK_a values, train/test labels and source references for all compounds studied in this work. Compounds labelled in green were chosen as the test set via a random 70/30 train:test split. Compounds shown in black correspond to the training set.

#	ID	Structure	pK_a	Source
**1**	**tk1**	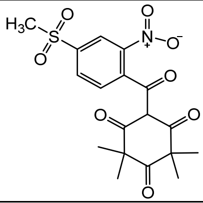	1.56	**Syngenta*
**2**	**tk3**	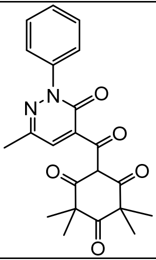	2.46	**Syngenta*
**3**	**tk4**	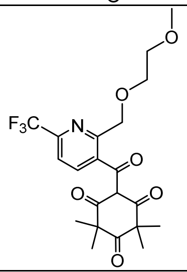	2.03	**Syngenta*
**4**	**tk5**	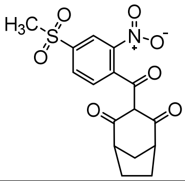	2.45	**Syngenta*
**5**	**tk6**	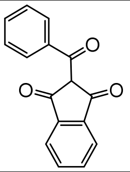	2.46	**Syngenta*
**6**	**tk7**	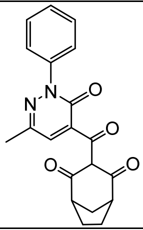	2.80	**Syngenta*
**7**	**tk8**	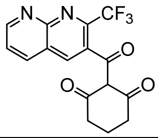	2.84	**Syngenta*
**8**	**tk9**	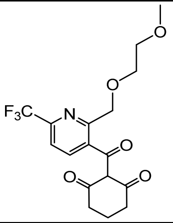	3.00	**Syngenta*

**9**	**tk10**	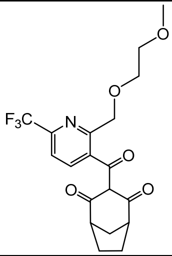	3.05	**Syngenta*
**10**	**tk11**	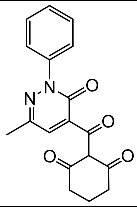	3.29	**Syngenta*
**11**	**tk12**	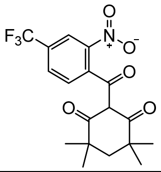	3.70	**Syngenta*
**12**	**tk13**	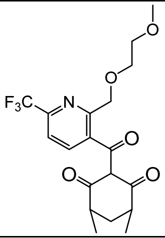	3.89	**Syngenta*
**13**	**tk14**	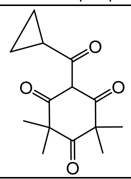	4.03	**Syngenta*
**14**	**tk15**	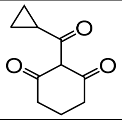	5.15	**Syngenta*
**15**	**tkn1**	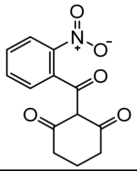	3.60	*Lee*[1]
**16**	**tkn2**	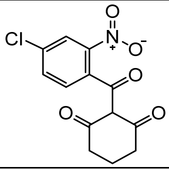	3.44	*Lee*[1]
**17**	**tkn3**	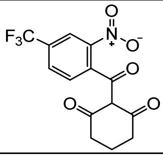	3.10	*Lee*[1]
**18**	**tkn4**	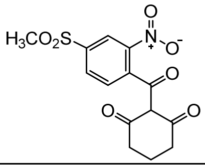	3.04	*Lee*[1]

**19**	**tkc1**	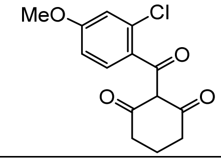	4.09	*Lee*[1]
**20**	**tkc2**	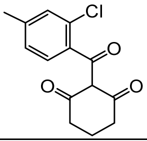	3.83	*Lee*[1]
**21**	**tkc3**	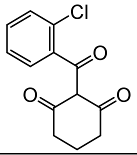	3.81	*Lee*[1]
**22**	**tkc4**	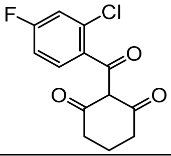	3.77	*Lee*[1]
**23**	**tkc5**	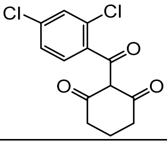	3.50	*Lee*[1]
**24**	**tkc6**	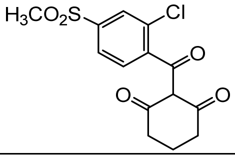	3.20	*Lee*[1]
**25**	**dk1**	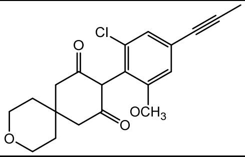	4.24	**Syngenta*
**26**	**dk2**	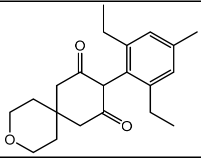	5.22	**Syngenta*
**27**	**dk3**	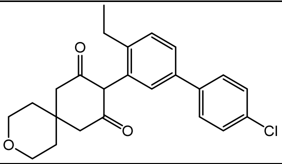	4.86	**Syngenta*
**28**	**dk4**	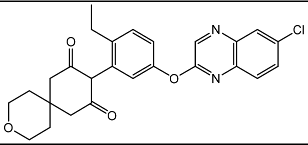	4.69	**Syngenta*
**29**	**dk5**	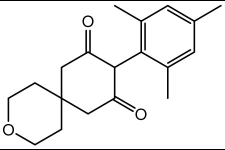	5.06	**Syngenta*
**30**	**dk6**	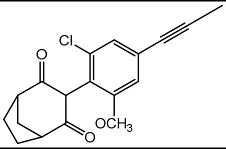	4.62	**Syngenta*

**31**	**dk7**	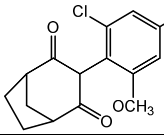	5.10	**Syngenta*
**32**	**dk8**	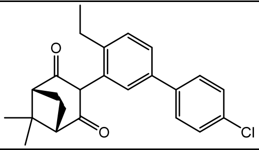	5.69	**Syngenta*
**33**	**dk9**	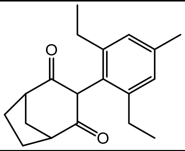	5.36	**Syngenta*
**34**	**dk10**	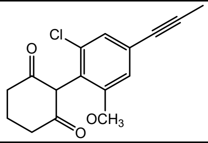	4.71	**Syngenta*
**35**	**dk11**	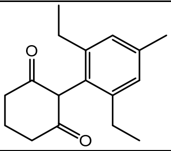	5.70	**Syngenta*
**36**	**dk12**	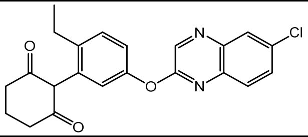	5.16	**Syngenta*
**37**	**dk13**	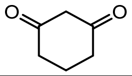	5.07	**this work*
**38**	**dk14**	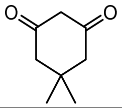	5.03	**this work*
**39**	**dk15**	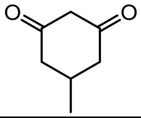	5.08	**this work*
**40**	**dk16**	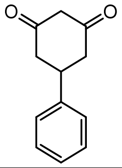	4.74	**this work*
**41**	**dk17**	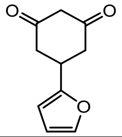	4.73	**this work*
**42**	**dk18**	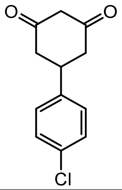	4.68	**this work*
**43**	**dk19**	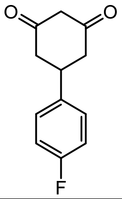	4.76	**this work*

**44**	**dk20**	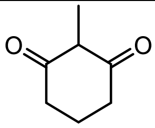	5.57	**this work*
**45**	**dk21**	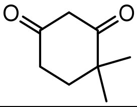	5.19	**this work*
**46**	**tk16**	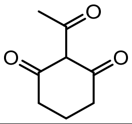	5.47	**this work*
**47**	**tk17**	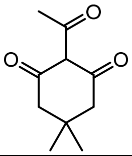	5.36	**this work*
**48**	**dk22**	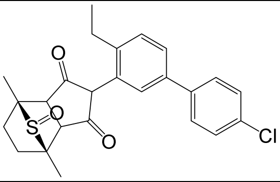	2.75	**Syngenta*
**49**	**dk23**	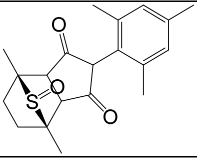	2.85	**Syngenta*
**50**	**dk24**	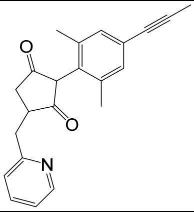	2.98	**Syngenta*
**51**	**dk25**	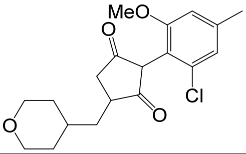	3.85	**Syngenta*
**52**	**dk26**	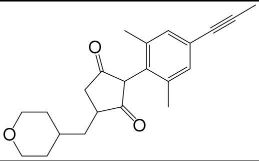	3.92	**Syngenta*
**53**	**dk27**	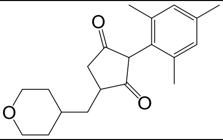	4.29	**Syngenta*
**54**	**dk28**	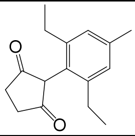	4.46	**Syngenta*
**55**	**dk29**	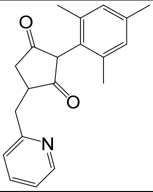	5.78	**Syngenta*

**56**	**tk18**	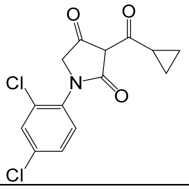	2.54	**Syngenta*
**57**	**tk19**	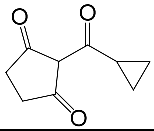	2.79	**Syngenta*
**58**	**Alloxydim (o1)**	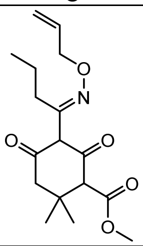	3.70[2]<br>3.81*	*Literature[2,3],*<br>**this work*
**59**	**Cycloxydim (o2)**	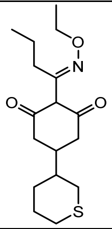	4.04[4]<br>4.51*	*literature[4],*<br>**this work*
**60**	**Butroxydim (o3)**	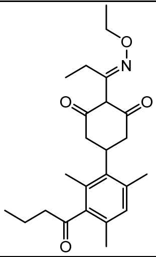	4.36[5]<br>4.34*	*literature[5],*<br>**this work*
**61**	**Sethoxydim (o4)**	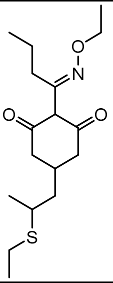	4.58[6]<br>4.48*	*literature[6],*<br>**this work*
**62**	**Clethodim (o5)**	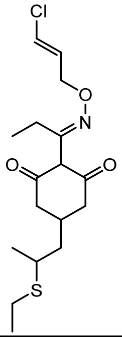	4.47 [7]	*literature[7],*

**63**	**Tepraloxydim (o6)**	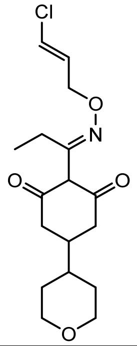	4.58[8] 4.30*	*literature[8], *this work*
**64**	**Tralkoxydim (o7)**	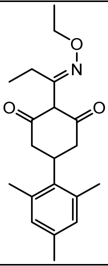	4.98[9] 4.35*	*literature[9], *this work*
**65**	**Profoxydim (o8)**	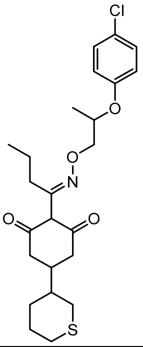	5.91[10] 4.82*	*literature[10], *this work*
**66**	**Tetracycline (tet1)**	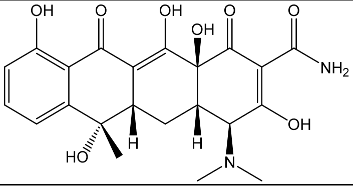	3.35	*literature[9]*
**67**	**4-Epianhydrotetracycline (tet2)**	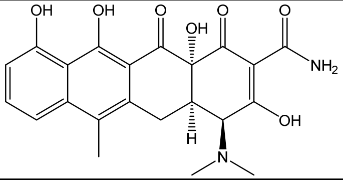	3.48	*literature[10]*
**68**	**Chlortetracycline (tet3)**	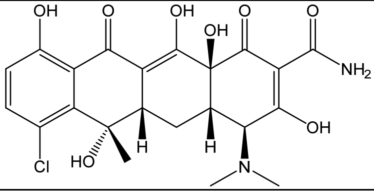	3.25	*literature[9]*
**69**	**Doxycycline (tet4)**	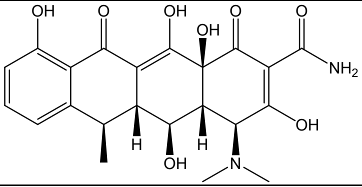	3.50	*literature[9]*
**70**	**Oxytetracycline (tet5)**	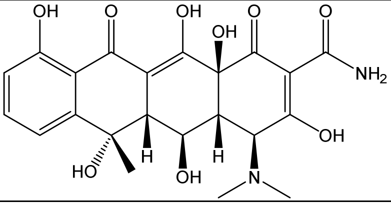	3.53	*Literature[9]*

**71**	**Demeclocycline (tet6)**	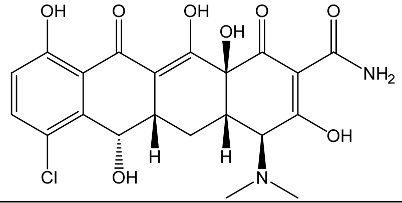	3.30	*literature[10]*

*See Supplementary Methods for methodological overview of experimental pK_a determination in *this work*.

Supplementary Note 1

Many of the sources for the herbicides **o1**-**o8** are not primary sources because they cannot be located (it is likely they were measured internally by the companies that developed them). However, many compounds feature in the "Pesticide Manual" produced by the British Crop Council. The literature values listed are validated by the current work (except in the case of profoxydim, **o8**) as there is good agreement between them and the new values measured here.

Details of experimental measurement of pK_a values for all compounds marked * in Supplementary Table 1 (i.e. "Syngenta" or "this work").

Experimental pK_a measurements were collected using a SiriusT3 instrument (Sirius Analytical Instruments, East Sussex, Great Britain), an automatic titration system incorporating *in situ* UV spectroscopy. The Sirius T3 is equipped with an Ag/AgCl double-junction reference electrode to monitor pH, a dip probe attached to a UV spectrophotometer, a stirrer, and all with automated volumetric titration capability. The Sirius T3 UV-metric pK_a measurement protocol measures the change in multi-wavelength absorbance in the UV region of the absorbance spectrum while the pH is titrated. Measurements were carried out at 25°C and constant ionic strength (0.01 M KCl) and UV absorbance data are collected from 160–760 nm while the 250–450 nm region is typically used for pK_a determinations.

Because of the low water solubility of the tested compounds, the titrations were carried out in a co-solvent (methanol): the method involves compound titration with three different methanol concentrations and the calculation of the pK_a by extrapolation using the Yasuda–Shedlovsky[12,13] equation. Two Sirius T3 computer programs, that is, Sirius T3 Control v1.1.3.0 and Sirius T3 Refine v1.1.3.0, were used to execute measurement protocols and analyse pH-dependent multi-wavelength spectra, respectively.

.

Supplementary Table 2. B3LYP/6-311G(d,p) solvent phase (CPCM) optimised bond lengths, T1 and T2 angles, **tkn** and **tkc** series' in the keto-enol *anti* tautomer (Fig. 3b), as well as experimental pK_a and log LD_{50} values taken from source[1]. Total wavefunction energies (optimized at B3LYP/6-311G(d,p) with CPCM) are shown in Supplementary Table 6.

ID	ortho	para	O-H	C-O	C=C	C-C	C=O	C-C	C=O	C-C	T1	T2	pK_a	log LD_{50}
**tkn1**	NO_2	H	0.96751	1.32948	1.37940	1.47843	1.22419	1.48137	1.21895	1.52129	17.74	72.54	3.60	2.91
**tkn2**	NO_2	Cl	0.96756	1.32845	1.37998	1.47821	1.22447	1.47925	1.21847	1.52261	17.11	72.70	3.44	1.62
**tkn3**	NO_2	CF_3	0.96759	1.32767	1.38058	1.47746	1.22477	1.47740	1.21857	1.52463	14.39	75.64	3.10	1.16
**tkn4**	NO_2	SO_3CH_3	0.96783	1.32682	1.38113	1.47707	1.22505	1.47563	1.21880	1.52590	10.90	77.11	3.04	1.12
**tkc1**	Cl	OMe	0.96590	1.34213	1.36190	1.46552	1.22756	1.51064	1.21818	1.49203	60.31	26.02	4.09	3.71
**tkc2**	Cl	Me	0.96617	1.33967	1.36484	1.46766	1.22706	1.50420	1.21686	1.50041	53.80	34.22	3.83	3.45
**tkc3**	Cl	H	0.96635	1.33778	1.36684	1.46912	1.22665	1.49980	1.21629	1.50562	49.25	40.37	3.81	2.92
**tkc4**	Cl	F	0.96628	1.33825	1.36626	1.46878	1.22680	1.50122	1.21616	1.50355	51.27	36.67	3.77	3.20
**tkc5**	Cl	Cl	0.96642	1.33632	1.36824	1.47000	1.22646	1.49737	1.21605	1.50705	46.76	40.44	3.50	2.26
**tkc6**	Cl	SO_2CH_3	0.96697	1.33210	1.37329	1.47307	1.22580	1.48791	1.21613	1.51549	36.64	51.65	3.20	1.86
NO_2 r^2			0.61	0.93	0.95	0.96	0.95	0.94	0.06	0.97				
Cl r^2			0.93	0.96	0.95	0.95	0.94	0.95	0.54	0.90				
full set r^2			0.69	0.77	0.68	0.59	0.50	0.76	0.07	0.76				
NO_2 r^2			0.43	0.85	0.82	0.70	0.81	0.84	0.37	0.80				
Cl r^2			0.86	0.92	0.90	0.90	0.91	0.90	0.53	0.86				
full set r^2			0.69	0.76	0.69	0.60	0.50	0.76	0.11	0.75				

Supplementary Table 3. B3LYP/6-311G(d,p) solvent phase (CPCM) optimised bond lengths of the **tkn** and **tkc** series' in the *endo* keto-enol *syn* tautomer (Fig. 3c), as well as experimental pK_a and log LD_{50} values taken from source[1]. Total wavefunction energies (optimized at B3LYP/6-311G(d,p) with CPCM) are shown in Supplementary Table 6.

ID	ortho	para	O-H	C-O	C=C	C-C	C=O	C-C	C=O	C-C	pK_a	log LD_{50}
**tkn1**	NO_2	H	1.00880	1.31073	1.39499	1.47308	1.22453	1.45572	1.24373	1.51195	3.60	2.91
**tkn2**	NO_2	Cl	1.00764	1.31028	1.39540	1.47320	1.22467	1.45421	1.24296	1.51308	3.44	1.62
**tkn3**	NO_2	CF_3	1.00618	1.31034	1.39482	1.47238	1.22470	1.45223	1.24229	1.51401	3.10	1.16
**tkn4**	NO_2	SO_3CH_3	1.00549	1.31024	1.39479	1.47215	1.22485	1.45148	1.24183	1.51494	3.04	1.12
**tkc1**	Cl	OMe	1.01376	1.30976	1.39799	1.47970	1.22254	1.45872	1.24996	1.49755	4.09	3.71
**tkc2**	Cl	Me	1.01420	1.30846	1.39936	1.47923	1.22249	1.45588	1.24865	1.50280	3.83	3.45
**tkc3**	Cl	H	1.01369	1.30795	1.39981	1.47881	1.22259	1.45426	1.24776	1.50569	3.81	2.92
**tkc4**	Cl	F	1.01308	1.30801	1.39980	1.47923	1.22259	1.45436	1.24768	1.50492	3.77	3.20
**tkc5**	Cl	Cl	1.01218	1.30770	1.39992	1.47871	1.22272	1.45275	1.24681	1.50683	3.50	2.26
**tkc6**	Cl	SO_2CH_3	1.01009	1.30740	1.39971	1.47728	1.22314	1.45010	1.24539	1.50955	3.20	1.86
NO_2 r^2			0.98	0.56	0.40	0.88	0.80	0.99	0.96	0.94		
Cl r^2			0.85	0.73	0.43	0.89	0.82	0.94	0.95	0.83		
full set r^2			0.75	0.05	0.25	0.56	0.52	0.77	0.77	0.78		
NO_2 r^2			0.86	0.89	0.06	0.50	0.78	0.87	0.88	0.85		
Cl r^2			0.81	0.70	0.42	0.84	0.78	0.90	0.92	0.81		
full set r^2			0.78	0.09	0.31	0.59	0.57	0.65	0.78	0.77		

Supplementary Table 4. B3LYP/6-311G(d,p) solvent phase (CPCM) optimised bond lengths of the **tkn** and **tkc** series' in the *exo* keto-enol *syn* tautomer (Fig. 3d), as well as experimental pK_a and log LD_{50} values taken from source[1]. Total wavefunction energies (optimized at B3LYP/6-311G(d,p) with CPCM) are shown in Supplementary Table 6.

ID	ortho	para	O-H	C-O	C=C	C-C	C=O	C-C	C=O	C-C	pK_a	log LD_{50}
**tkn1**	NO_2	H	1.25073	1.45792	1.48178	1.22264	1.40101	1.31071	1.02895	1.48335	3.60	2.91
**tkn2**	NO_2	Cl	1.24991	1.45883	1.48170	1.22241	1.39752	1.30897	1.02894	1.48926	3.44	1.62
**tkn3**	NO_2	CF_3	1.24928	1.45946	1.48152	1.22245	1.39544	1.30820	1.02849	1.49215	3.10	1.16
**tkn4**	NO_2	SO_3CH_3	1.24922	1.45980	1.48192	1.22243	1.39556	1.30836	1.02890	1.49124	3.04	1.12
**tkc1**	Cl	OMe	1.24857	1.46010	1.48143	1.22250	1.39355	1.30794	1.02808	1.49285	4.09	3.71
**tkc2**	Cl	Me	1.24795	1.46042	1.48070	1.22268	1.39131	1.30731	1.02808	1.49468	3.83	3.45
**tkc3**	Cl	H	1.25092	1.45403	1.47484	1.22469	1.39703	1.30408	1.03193	1.49681	3.81	2.92
**tkc4**	Cl	F	1.25030	1.45507	1.47530	1.22470	1.39552	1.30385	1.03223	1.49720	3.77	3.20
**tkc5**	Cl	Cl	1.24995	1.45487	1.47460	1.22474	1.39390	1.30359	1.03173	1.49825	3.50	2.26
**tkc6**	Cl	SO_2CH_3	1.24681	1.45350	1.47525	1.22110	1.39540	1.30110	1.03204	1.49870	3.20	1.86
NO_2 r^2			0.89	0.88	0.01	0.93	0.93	0.95	0.14	0.93		
Cl r^2			0.40	0.02	0.02	0.20	0.16	0.18	0.01	0.24		
full set r^2			0.35	0.19	0.43	0.01	0.54	0.65	0.23	0.75		
NO_2 r^2			0.84	0.74	0.21	0.44	0.85	0.83	0.71	0.85		
Cl r^2			0.22	0.06	0.07	0.08	0.07	0.24	0.07	0.30		
full set r^2			0.27	0.24	0.48	0.03	0.44	0.67	0.30	0.72		

Supplementary Table 5. B3LYP/6-311G(d,p) solvent phase (CPCM) optimised bond lengths of the **tkn** and **tkc** series' in the triketo tautomer (Fig. 3a) as well as experimental pK_a and log LD_{50} values taken from source[1]. Total wavefunction energies (optimized at B3LYP/6-311G(d,p) with CPCM) are shown in Supplementary Table 6.

ID	ortho	para	C=O	C-C	C-H	C-C	C=O	C-C	C=O	C-C	pK_a	log LD_{50}
**tkn1**	NO_2	H	1.21000	1.53900	1.10042	1.54009	1.21055	1.54168	1.21696	1.48718	3.60	2.91
**tkn2**	NO_2	Cl	1.21001	1.53882	1.10046	1.54088	1.21055	1.53901	1.21422	1.49505	3.44	1.62
**tkn3**	NO_2	CF_3	1.21007	1.53890	1.10026	1.54156	1.21054	1.53753	1.21260	1.50014	3.10	1.16
**tkn4**	NO_2	SO_3CH_3	1.20995	1.53918	1.10024	1.54124	1.21061	1.53780	1.21294	1.49822	3.04	1.12
**tkc1**	Cl	OMe	1.20987	1.53981	1.10020	1.54110	1.21075	1.53599	1.21144	1.50298	4.09	3.71
**tkc2**	Cl	Me	1.20983	1.53984	1.09979	1.54297	1.21047	1.53299	1.20920	1.51130	3.83	3.45
**tkc3**	Cl	H	1.20860	1.54072	1.09720	1.55344	1.20881	1.52930	1.20951	1.51534	3.81	2.92
**tkc4**	Cl	F	1.20873	1.54108	1.09727	1.55416	1.20876	1.52845	1.20864	1.51683	3.77	3.20
**tkc5**	Cl	Cl	1.20882	1.54066	1.09707	1.55400	1.20863	1.52744	1.20843	1.51786	3.50	2.26
**tkc6**	Cl	SO_2CH_3	1.20890	1.54064	1.09705	1.55413	1.20862	1.52690	1.20800	1.51874	3.20	1.86
NO_2 r^2			0.00	0.00	0.32	0.83	0.16	0.93	0.92	0.89		
Cl r^2			0.00	0.00	0.04	0.04	0.05	0.19	0.43	0.21		
full set r^2			0.36	0.55	0.46	0.45	0.39	0.74	0.84	0.78		
NO_2 r^2			0.17	0.00	0.65	0.94	0.01	0.85	0.87	0.90		
Cl r^2			0.01	0.05	0.10	0.10	0.12	0.25	0.44	0.24		
full set r^2			0.40	0.61	0.51	0.51	0.45	0.75	0.80	0.76		

Supplementary Table 6. B3LYP/6-311G(d,p) /CPCM total energies in Hartree for each tautomeric form of the **tkn** and **tkc** series.

ID	keto-enol (endo) anti (***b***)	keto-enol (endo) syn (***c***)	keto-enol (exo) syn (***d***)	Diketo (***a***)
**tkn1**	-933.0286676	-933.0464026	-933.0431372	-933.02545
**tkn2**	-1392.647259	-1392.66457	-1392.660993	-1392.6435
**tkn3**	-1270.165215	-1270.182164	-1270.178273	-1270.161
**tkn4**	-1776.065033	-1776.081334	-1776.078701	-1776.0611
**tkc1**	-1302.650311	-1302.664479	-1302.66302	-1302.6476
**tkc2**	-1227.421956	-1227.437151	-1227.435437	-1227.4191
**tkc3**	-1188.093305	-1188.108998	-1188.107143	-1188.0901
**tkc4**	-1287.356209	-1287.371518	-1287.369568	-1287.353
**tkc5**	-1647.71318	-1647.728783	-1647.726659	-1647.7097
**tkc6**	-1776.065033	-1776.081334	-1776.078701	-1776.0611

Supplementary Note 2

The origin of bond length variation with pK_a for 2-NO_2 (tkn) and 2-Cl (tkc) substituted triketones.

Initial analysis of the internal validation statistics for the full set of 10 **tkn** and **tkc** compounds in total, reveals, in each state, no r^2 values above 0.90 for any plot of bond length ***i*** to ***viii*** *vs* pK_a. However, correlations increase for each bond length when the set is split into two subsets. Intuitively, the full set is split according to the 2-substituent type, into one set consisting of compounds **tkn1** – **tkn4**, and another set of compounds **tkc1** – **tkc6**. Whilst Cl atoms are electron-withdrawing through σ-bonds due to their electronegativity, the electron-withdrawing effect of an NO_2 group occurs not only due to the electronegativity of the nitrogen atom (i.e. σ-effects) but also due to a π-withdrawal effect. Furthermore, the electron-withdrawing capacity of Cl is reduced by its positive mesomeric effect, i.e., π-donation into the ring. Overall, by a comparison of the strength of the correlation of all bond lengths with pK_a across each tautomer, the keto-enol *anti* tautomer **b** (Figure 3A of the main text) comes out on top. Bond lengths and statistics for all tautomers **a**-**d** can be found in Tables S2 to S5.

For both subsets, the trend in the bond variation of O-H ***i***, C-O ***ii*** and C=C ***iii*** with pK_a is such that more acidic compounds have longer O-H and C=C bonds but shorter C-O distances. These observations fit with the intuition that a longer, weaker O-H bond should exhibit an increased propensity for dissociation. However, there are some inconsistencies between the two sets when we now consider the remaining bonds. Firstly, considering the 2-NO_2 set (still as tautomer **b** i.e., the *anti* keto-enol form), the more acidic species are found to have shorter C-C ***iv*** and ***vi*** bonds, and longer C=O ***v*** bonds, suggesting that the delocalisation occurs across the whole triketone system. Evidence for this assertion lies in the co-planar orientation of the keto-enol moiety with the *exo* carbonyl group: **tkn1** – **tkn4** have an average C^1=C^2-C^3=O^4 dihedral angle **T1** of 15° (Figure 3B of the main text). However, the plot of bond ***vii*** *vs* pK_a does not provide corroboration of the above assertion because, rather than showing the expected negative correlation, the two variables are completely uncorrelated (r^2= 0.06). For these same four compounds, the *exo* carbonyl group is almost orthogonal to the phenyl ring: the C^6=C^5-C^3=O^4 torsional angle **T2** has an average value of 75°. This orthogonality is indicative of negligible conjugation of the 2-Ac-1,3-CHD group with the aromatic ring. It should also be noted that the magnitude of **T1** and **T2** dihedral angles also correlate with pK_a, with r^2 values 0.84 and 0.92, respectively. That is to say, the more co-planar the two C=O and single C-O moieties of the triketone fragment are, the lower the pK_a.

Now considering the **tkc** subset, the average **T1** and **T2** angles are found to be ~50°, and ~38°, respectively. This preferred geometry is indicative of an increase in the degree of conjugation with the aromatic ring. This assertion is corroborated by the fact that **T1** and **T2** values also correlate to aqueous pK_a values, with r^2 values of 0.95 (with a positive gradient) and 0.89 (with a negative gradient),

respectively i.e., as the *exo* carbonyl group of the **tkc** series becomes more in-plane with benzene, these compounds become more acidic. As is illustrated in Figure 3B of the main text, the C-C ***iv*** and C=O ***v*** bonds of the 2-Cl subset are also found to show opposing trends with pK_a when compared to the same bonds of the 2-NO_2 analogues. A longer C-C bond and shorter C=O bond is again indicative of less conjugation with the keto group. Finally, whereas the slope of the line-of-best-fit for both subsets is negative for bond distance ***viii,*** i.e., the C-C bond linking to the phenyl group, those of the 2-Cl series are consistently shorter those of the 2-NO_2 set. From this observation, it may again be inferred that there is a greater degree of conjugation into the phenyl ring through the *exo* carbonyl for **C1** to **C6**, as the C-C ***viii*** bond gains more double bond character.

In order to further explain the above observations, an IQA analysis was performed on the B3LYP/6-311G(d,p) calculated wavefunctions of all 10 derivatives. The images shown in Fig.S1 (a) and (b) were generated using the wavefunction of the global minimum geometry of the 2-NO_2 and 2-Cl,4-OMe derivatives with MORFI. The atomic basins are shown, and all Bond Critical Points (BCPs) are marked in purple. The first obvious difference between Fig. S1 (a) and (b) is that BCPs are found between O^8 and C^5 (blue arrow), and between O^9 and O^4 (green arrow) in (a), whereas they are absent in (b). Whilst the presence of a BCP does not mean that there is a bonding interaction *per se*, it does suggest that the interaction should be looked at in more detail. Inspection of the full E_{IQA} interaction energy between O^8 and C^5 for both types of compound reveals that it is generally -25 kJ mol^{-1} more negative for the four **tkn** (2-NO_2) derivatives on average compared to the six 2-Cl compounds. Looking at the V_{xc} contribution to this E_{IQA} value, the 2-NO_2 derivatives are found to be -19 kJ mol^{-1} more negative on average than the average value for the 2-Cl subset. A substantial increase in the exchange interaction between the o^8 atom and the *ipso* carbon of the phenyl ring explains the lengthening of the C=O ***v*** bond. This is because an increase in delocalisation of electrons between O^8 and C^5 means there are fewer electrons available to partake in delocalisation between the constituent atoms of the C=O bond. Correspondingly, as the bond length increases, the V_{xc} value for the C=O becomes more positive.

Visual inspection of the Highest Occupied Molecular Orbital (HOMO) of compounds of each type (i.e. t**kn** or **tkc**), reveals evidence of orbital overlap between the exo carbonyl group and the phenyl ring for the **tkc** species, which is absent in the **tkc** analogue. This is shown in Supplementary Figure 1 (c) and (d), for compounds **tkn3** and **tkc6**, and may be taken as further corroboration of the relative increase conjugation between the phenyl ring and exo-carbonyl group for the **tkc** analogues in this tautomeric state and conformation.

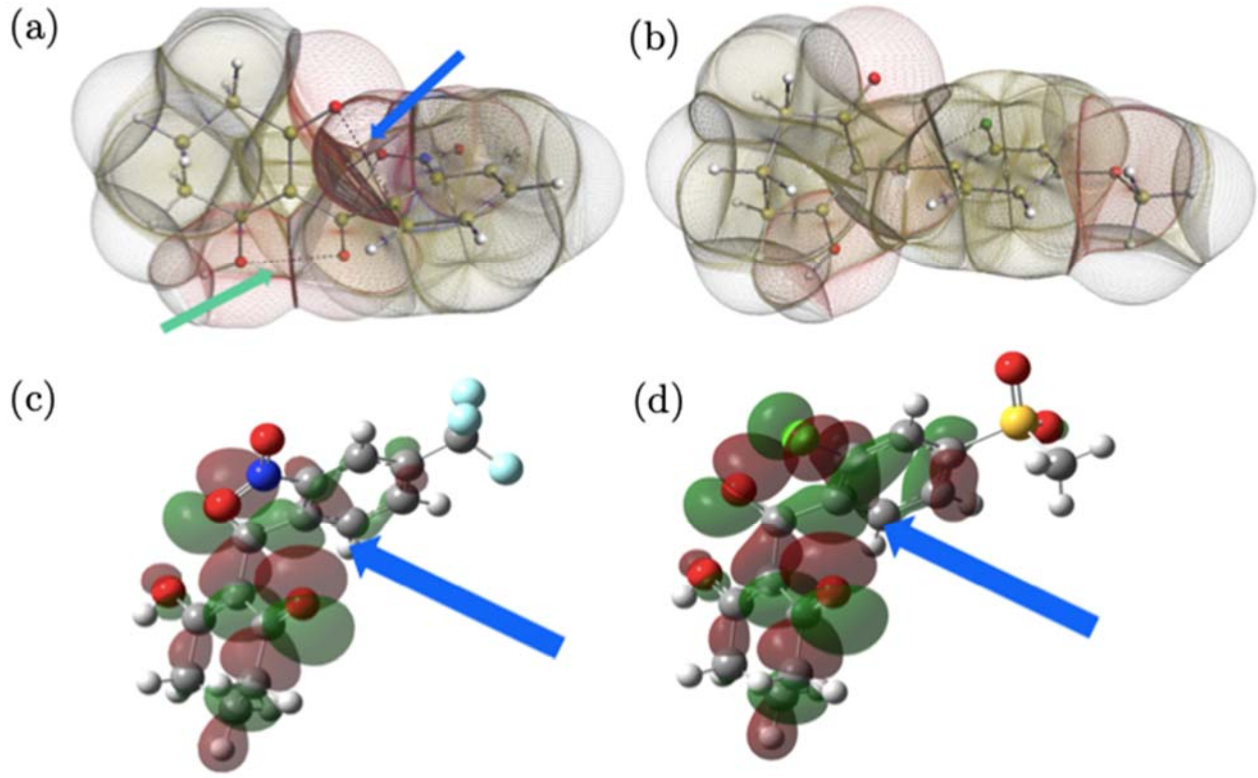

Supplementary Figure 1. (a) The atomic basins of 2-(2-NO_2-phenyl)-1,3-cyclohexanedione and the bond critical points (purple) between O^8 and C^5 (blue arrow) and O^9 and O^4 (green arrow). (b) The atomic basins of 2-(2-Cl,4-OMe-phenyl)-1,3-cyclohexanedione, showing that the bond critical points highlighted in (a) are now absent. (c) HOMO of **tkn3**, showing a lack of orbital overlap between the *exo* carbonyl moiety and the phenyl ring. (d) HOMO of **tkc6**, showing possible orbital overlap due to increase in co-planarity between the exo carbonyl group and the phenyl ring.

Supplementary Table 7. Bond lengths (***i***-***v***) and pK_a values for the most stable conformation identified at B3LYP/6-311G(d,p) in CPCM for all compounds studied in this work, whilst keeping the keto-enol *anti* state of the 1,3-CHD or 1,3-CPD group.

RING SIZE	*type*	ID	O-H (*i*)	C-O (*ii*)	C=C (*iii*)	C-C (*iv*)	C=O (*v*)	pK_a
6	*triketone*	**tk1**	0.96800	1.32333	1.37699	1.47240	1.22232	1.56
6	*triketone*	**tk3**	0.96518	1.34120	1.35956	1.45886	1.22561	2.46
6	*triketone*	**tk4**	0.96680	1.33209	1.36960	1.46311	1.22589	2.03
6	*triketone*	**tk5**	0.96779	1.32533	1.38229	1.47782	1.22515	2.45
5	*triketone*	**tk6**	0.96709	1.32108	1.37846	1.48605	1.21694	2.46
6	*triketone*	**tk7**	0.96673	1.33374	1.37241	1.47358	1.22622	2.80
6	*triketone*	**tk8**	0.96685	1.33205	1.37322	1.47363	1.22567	2.84
6	*triketone*	**tk9**	0.96689	1.33326	1.37383	1.47470	1.22618	3.00
6	*triketone*	**tk10**	0.96725	1.33073	1.37681	1.47736	1.22597	3.05
6	*triketone*	**tk11**	0.96654	1.33588	1.37030	1.47056	1.22675	3.29
6	*triketone*	**tk12**	0.96593	1.33457	1.37145	1.47077	1.22675	3.70
6	*triketone*	**tk13**	0.96710	1.33574	1.37197	1.47198	1.22755	3.89
6	*triketone*	**tk14**	0.96524	1.34224	1.35959	1.45910	1.22530	4.03
6	*triketone*	**tk15**	0.96624	1.33919	1.36785	1.46930	1.22762	5.15
6	*triketone*	**tkn1**	0.96751	1.32948	1.37940	1.47843	1.22419	3.60
6	*triketone*	**tkn2**	0.96756	1.32845	1.37998	1.47821	1.22447	3.44
6	*triketone*	**tkn3**	0.96759	1.32767	1.38058	1.47746	1.22477	3.10
6	*triketone*	**tkn4**	0.96783	1.32682	1.38113	1.47707	1.22505	3.04
6	*triketone*	**tkc1**	0.96590	1.34213	1.36190	1.46552	1.22756	4.09
6	*triketone*	**tkc2**	0.96617	1.33967	1.36484	1.46766	1.22706	3.83
6	*triketone*	**tkc3**	0.96635	1.33778	1.36684	1.46912	1.22665	3.81
6	*triketone*	**tkc4**	0.96628	1.33825	1.36626	1.46878	1.22680	3.77
6	*triketone*	**tkc5**	0.96642	1.33632	1.36824	1.47000	1.22646	3.50
6	*triketone*	**tkc6**	0.96697	1.33210	1.37329	1.47307	1.22580	3.20
6	*diketone*	**dk1**	0.96571	1.34718	1.35746	1.46509	1.22678	4.24
6	*diketone*	**dk2**	0.96565	1.35126	1.35795	1.46564	1.22875	5.22
6	*diketone*	**dk3**	0.96562	1.34926	1.35904	1.46562	1.22819	4.86
6	*diketone*	**dk4**	0.96568	1.34888	1.35820	1.46501	1.22806	4.69
6	*diketone*	**dk5**	0.96566	1.35075	1.35814	1.46599	1.22845	5.06
6	*diketone*	**dk6**	0.96565	1.34511	1.35991	1.46785	1.22746	4.62
6	*diketone*	**dk7**	0.96557	1.34580	1.35988	1.46770	1.22770	5.10
6	*diketone*	**dk8**	0.96566	1.34814	1.35807	1.46878	1.22861	5.69
6	*diketone*	**dk9**	0.96565	1.35006	1.36031	1.47126	1.22843	5.36
6	*diketone*	**dk10**	0.96574	1.34688	1.35905	1.46693	1.22712	4.71
6	*diketone*	**dk11**	0.96556	1.35083	1.35952	1.46733	1.22908	5.70
6	*diketone*	**dk12**	0.96575	1.34919	1.36028	1.46845	1.22786	5.16
6	*diketone*	**dk13**	0.96556	1.34751	1.35456	1.45373	1.22924	5.07
6	*diketone*	**dk14**	0.96549	1.34799	1.35303	1.45156	1.22962	5.03
6	*diketone*	**dk15**	0.96559	1.34771	1.35395	1.45284	1.22948	5.08
6	*diketone*	**dk16**	0.96575	1.34686	1.35374	1.45220	1.22883	4.74
6	*diketone*	**dk17**	0.96572	1.34642	1.35375	1.45189	1.22863	4.73

6	*diketone*	**dk18**	0.96572	1.34651	1.35370	1.45205	1.22855	4.68
6	*diketone*	**dk19**	0.96573	1.34670	1.35372	1.45215	1.22872	4.76
6	*diketone*	**dk20**	0.96523	1.35340	1.35899	1.46392	1.23016	5.57
6	*diketone*	**dk21**	0.96564	1.34788	1.35314	1.45446	1.22931	5.19
6	*triketone*	**tk16**	0.96609	1.34008	1.36735	1.47021	1.22799	5.47
6	*triketone*	**tk17**	0.96600	1.33993	1.36644	1.46871	1.22823	5.36
5	*diketone*	**dk22**	0.96636	1.33521	1.35886	1.46315	1.22049	2.75
5	*diketone*	**dk23**	0.96589	1.33851	1.35731	1.46313	1.22163	2.85
5	*diketone*	**dk24**	0.96656	1.33444	1.35970	1.45805	1.22150	2.98
5	*diketone*	**dk25**	0.96626	1.33565	1.35625	1.46174	1.22195	3.85
5	*diketone*	**dk26**	0.96638	1.33731	1.35851	1.46357	1.22340	3.92
5	*diketone*	**dk27**	0.96626	1.33830	1.35821	1.46299	1.22394	4.29
5	*diketone*	**dk28**	0.96621	1.33856	1.35765	1.46438	1.22325	4.46
5	*diketone*	**dk29**	0.96664	1.33432	1.35983	1.45792	1.22494	5.78
5	*triketone*	**tk18**	0.96779	1.32326	1.36401	1.48929	1.22142	2.54
5	*triketone*	**tk19**	0.96803	1.32126	1.37481	1.47497	1.22104	2.79
6	*dim*	**o1**	0.96601	1.34263	1.36055	1.45890	1.22687	3.81
6	*dim*	**o2**	0.96574	1.34523	1.36022	1.46202	1.22898	4.51
6	*dim*	**o3**	0.96587	1.34538	1.36151	1.46418	1.22865	4.34
6	*dim*	**o4**	0.96579	1.34519	1.36139	1.46430	1.22884	4.48
6	*dim*	**o4**	0.96580	1.34422	1.36149	1.46428	1.22862	4.47
6	*dim*	**o6**	0.96578	1.34531	1.36077	1.46292	1.22894	4.30
6	*dim*	**o7**	0.96578	1.34596	1.36158	1.46565	1.22863	4.35
6	*dim*	**o8**	0.96592	1.34500	1.36006	1.46183	1.22890	4.82
6	*triketone*	**tet1**	0.96685	1.33124	1.37383	1.45361	1.23080	3.35
6	*triketone*	**tet2**	0.96650	1.33394	1.36917	1.47136	1.22588	3.48
6	*triketone*	**tet3**	0.96657	1.33147	1.37335	1.45366	1.23041	3.25
6	*triketone*	**tet4**	0.96685	1.33297	1.37031	1.47032	1.22544	3.50
6	*triketone*	**tet5**	0.96674	1.33313	1.37024	1.46821	1.22630	3.53
6	*triketone*	**tet6**	0.96684	1.33120	1.37368	1.45303	1.23065	3.30

Supplementary Table 8. The ID for each combination of features of the 5 common to the keto-enol fragment.

Model ID	Features				# features
**1**	O-H				1
**2**	C-O				1
**3**	C=C				1
**4**	CC				1
**5**	C=O				1
**6**	O-H	C-O			2
**7**	O-H	C=C			2
**8**	O-H	CC			2
**9**	O-H	C=O			2
**10**	C-O	C=C			2
**11**	C-O	CC			2
**12**	C-O	C=O			2
**13**	C=C	CC			2
**14**	C=C	C=O			2
**15**	CC	C=O			2
**16**	O-H	C-O	C=C		3
**17**	O-H	C-O	CC		3
**18**	O-H	C-O	C=O		3
**19**	O-H	C=C	CC		3
**20**	O-H	C=C	C=O		3
**21**	O-H	CC	C=O		3
**22**	C-O	C=C	CC		3
**23**	C-O	C=C	C=O		3
**24**	C-O	CC	C=O		3
**25**	C=C	CC	C=O		3
**26**	O-H	C-O	C=C	CC	4
**27**	O-H	C-O	C=C	C=O	4
**28**	O-H	C-O	CC	C=O	4
**29**	O-H	C=C	CC	C=O	4
**30**	C-O	C=C	CC	C=O	4
**31**	ALL				5

Supplementary Table 9. 7-fold CV RMSEE, MAE, and R^2 score for PLS regression using each combination of the 5 bond lengths of the keto-enol fragment. The optimal model based on RMSEE is marked in red.

MODEL	R2 SCORE	RMSEE	MAE	LV
1	0.554	0.746	0.604	1
2	0.712	0.567	0.418	1
3	0.378	0.887	0.652	1
4	0.155	1.073	0.879	1
5	0.374	0.898	0.732	1
6	0.662	0.629	0.486	1
7	0.511	0.790	0.600	1
8	0.467	0.826	0.648	1
9	0.625	0.670	0.554	1
10	0.611	0.687	0.501	1
11	0.606	0.692	0.518	1
12	0.691	0.599	0.461	1
13	0.339	0.925	0.698	1
14	0.600	0.695	0.545	1
15	0.353	0.928	0.728	1
16	0.722	0.577	0.466	2
17	0.702	0.606	0.511	2
18	0.723	0.588	0.469	2
19	0.559	0.812	0.664	2
20	0.639	0.668	0.541	2
21	0.689	0.655	0.575	2
22	0.706	0.609	0.489	2
23	0.707	0.592	0.453	2
24	0.756	0.539	0.417	2
25	0.692	0.663	0.515	2
26	0.735	0.579	0.476	3
27	0.738	0.578	0.461	3
28	0.754	0.539	0.435	3
29	0.728	0.600	0.507	3
**30**	**0.759**	**0.530**	**0.410**	**3**
31	0.758	0.543	0.446	3

Supplementary Table 10. 7-fold CV RMSEE, MAE, and R^2 score for RF regression using each combination of the 5 bond lengths of the keto-enol fragment. The optimal model based on RMSEE is marked in red.

MODEL	R2	RMSEE	MAE	max dept	n est
**1**	0.809	0.700	0.583	3	25
**2**	0.948	0.623	0.518	6	10
**3**	0.649	0.955	0.763	3	50
**4**	0.632	0.962	0.815	3	500
**5**	0.870	0.734	0.605	4	10
**6**	0.958	0.647	0.546	6	500
**7**	0.879	0.763	0.634	4	25
**8**	0.919	0.727	0.588	5	50
**9**	0.960	0.608	0.538	6	25
**10**	0.944	0.650	0.533	6	10
**11**	0.948	0.599	0.497	5	25
**12**	0.967	0.573	0.463	6	100
**13**	0.884	0.926	0.720	6	75
**14**	0.957	0.618	0.530	5	500
**15**	0.931	0.727	0.543	6	25
**16**	0.957	0.640	0.529	6	100
**17**	0.949	0.620	0.523	5	25
**18**	0.965	0.577	0.475	6	50
**19**	0.905	0.760	0.632	4	100
**20**	0.949	0.695	0.594	5	10
**21**	0.954	0.622	0.513	5	25
**22**	0.900	0.641	0.533	3	10
**23**	0.954	0.596	0.493	5	25
**24**	**0.959**	**0.567**	**0.457**	**6**	**25**
**25**	0.953	0.634	0.514	5	50
**26**	0.956	0.659	0.552	6	75
**27**	0.953	0.600	0.489	5	25
**28**	0.960	0.573	0.459	6	25
**29**	0.957	0.646	0.526	6	50
**30**	0.916	0.628	0.501	3	10
**31**	0.940	0.648	0.515	4	10

Supplementary Table 11. 7-fold CV RMSEE, MAE, and R^2 score for SV regression with a linear kernel using each combination of the 5 bond lengths of the keto-enol fragment. The optimal model based on RMSEE is marked in red.

MODEL	R2	RMSEE	MAE	C	epsilon
1	0.010	1.110	0.960	1000	1
2	0.695	0.582	0.432	1000	0.0001
3	0.364	0.827	0.614	1000	0.1
4	0.139	1.021	0.832	1000	0.1
5	0.109	1.025	0.888	1000	1
6	0.695	0.582	0.432	1000	0.0005
7	0.367	0.826	0.614	1000	0.1
8	0.150	1.018	0.831	1000	0.1
9	0.114	1.023	0.885	1000	1
10	0.668	0.616	0.454	1000	0.01
11	0.687	0.590	0.442	1000	0.0001
**12**	**0.703**	**0.573**	**0.428**	**1000**	**0.1**
13	0.368	0.857	0.643	1000	0.0001
14	0.459	0.785	0.593	1000	0.01
15	0.219	0.961	0.784	1000	0.01
16	0.668	0.616	0.454	1000	0.01
17	0.687	0.590	0.442	1000	0.0001
**18**	0.702	0.574	0.429	1000	0.1
19	0.372	0.855	0.642	1000	0.0001
20	0.462	0.785	0.594	1000	0.01
21	0.230	0.960	0.785	1000	0.01
22	0.669	0.630	0.466	1000	0.0005
23	0.676	0.609	0.453	1000	0.0001
24	0.708	0.590	0.446	1000	0.1
25	0.466	0.817	0.621	1000	0.001
26	0.669	0.631	0.467	1000	0.0001
27	0.677	0.609	0.453	1000	0.0001
28	0.708	0.590	0.447	1000	0.1
29	0.469	0.817	0.622	1000	0.0001
30	0.688	0.619	0.469	1000	0.01
31	0.688	0.619	0.469	1000	0.01

Supplementary Table 12. 7-fold CV RMSEE, MAE, and R^2 score for SV regression with an RBF kernel using each combination of the 5 bond lengths of the keto-enol fragment. The optimal model based on RMSEE is marked in red.

MODEL	R2	RMSEE	MAE	C	epsilon	gamma
1	0.181	1.030	0.891	1000	0.1	5
2	0.709	0.551	0.400	1000	0.01	5
3	0.367	0.822	0.601	1000	0.01	5
4	0.125	1.010	0.798	1000	0.1	5
5	0.361	0.946	0.802	1000	0.1	5
6	0.709	0.550	0.400	1000	0.01	5
7	0.387	0.815	0.597	1000	0.01	5
8	0.209	0.956	0.756	1000	0.05	5
9	0.420	0.854	0.726	1000	0.5	5
10	0.723	0.546	0.406	1000	0.05	5
11	0.712	0.558	0.408	1000	0.0001	3
12	0.717	0.546	0.403	1000	0.0001	5
13	0.373	0.854	0.624	1000	0.01	1
14	0.557	0.725	0.554	1000	0.1	5
15	0.346	0.880	0.706	1000	0.0001	5
16	0.723	0.547	0.405	1000	0.05	5
17	0.712	0.558	0.408	1000	0.0001	3
18	0.718	0.547	0.403	1000	0.0001	5
19	0.396	0.852	0.626	1000	0.0005	5
20	0.560	0.727	0.558	1000	0.1	5
21	0.413	0.858	0.700	1000	0.05	5
22	0.731	0.551	0.411	1000	0.0001	5
23	0.723	0.554	0.415	1000	0.005	3
**24**	**0.753**	**0.533**	**0.401**	**1000**	**0.1**	**5**
25	0.652	0.666	0.492	1000	0.0005	5
26	0.731	0.550	0.409	1000	0.0001	5
27	0.723	0.553	0.414	1000	0.005	3
28	0.753	0.534	0.402	1000	0.1	5
29	0.655	0.665	0.489	1000	0.05	5
30	0.755	0.533	0.405	1000	0.05	5
31	0.756	0.533	0.405	1000	0.05	5

Supplementary Table 13. 7-fold CV RMSEE, MAE, and R^2 score for GP regression with an RBF kernel using each combination of the 5 bond lengths of the keto-enol fragment. The optimal models based on RMSEE are marked in red.

	MAE	RMSEE	R2
1	0.69	0.84	-2.15
2	0.56	0.76	-1.58
3	0.70	0.85	-2.25
4	0.64	0.72	-1.37
**5**	**0.30**	**0.49**	**-0.06**
6	0.56	0.65	-0.88
7	0.66	0.86	-2.36
8	0.64	0.72	-1.37
**9**	**0.30**	**0.49**	**-0.06**
10	0.54	0.70	-1.20
11	0.56	0.65	-0.88
12	0.52	0.58	-0.50
13	0.70	0.83	-2.12
14	0.35	0.50	-0.11
15	0.28	0.51	-0.16
16	0.64	0.88	-2.47
17	0.56	0.74	-1.46
18	0.54	0.64	-0.85
19	0.68	0.85	-2.29
20	0.35	0.50	-0.11
21	0.36	0.56	-0.42
22	0.62	0.82	-2.00
23	0.44	0.65	-0.92
24	0.44	0.62	-0.74
25	0.33	0.51	-0.19
26	0.54	0.70	-1.20
27	0.33	0.51	-0.15
28	0.32	0.55	-0.35
29	0.33	0.51	-0.19
30	0.33	0.51	-0.15
31	0.33	0.51	-0.15

Supplementary Figure 2. For the series **o1**-**o8**: Marvin predictions (no consideration of tautomers/resonance), literature experimental values ("Lit Exp"), our newly measured experimental values ("Our Exp") and AIBL predictions made using the C-O bond model, i.e. $pK_a = 98.38*r(C\text{-}O)-127.71$. An asterisk (*) denotes predictions for compounds that feature in the test set, i.e. are external predictions. Those without asterisks feature in the training set used to formulate the model itself.

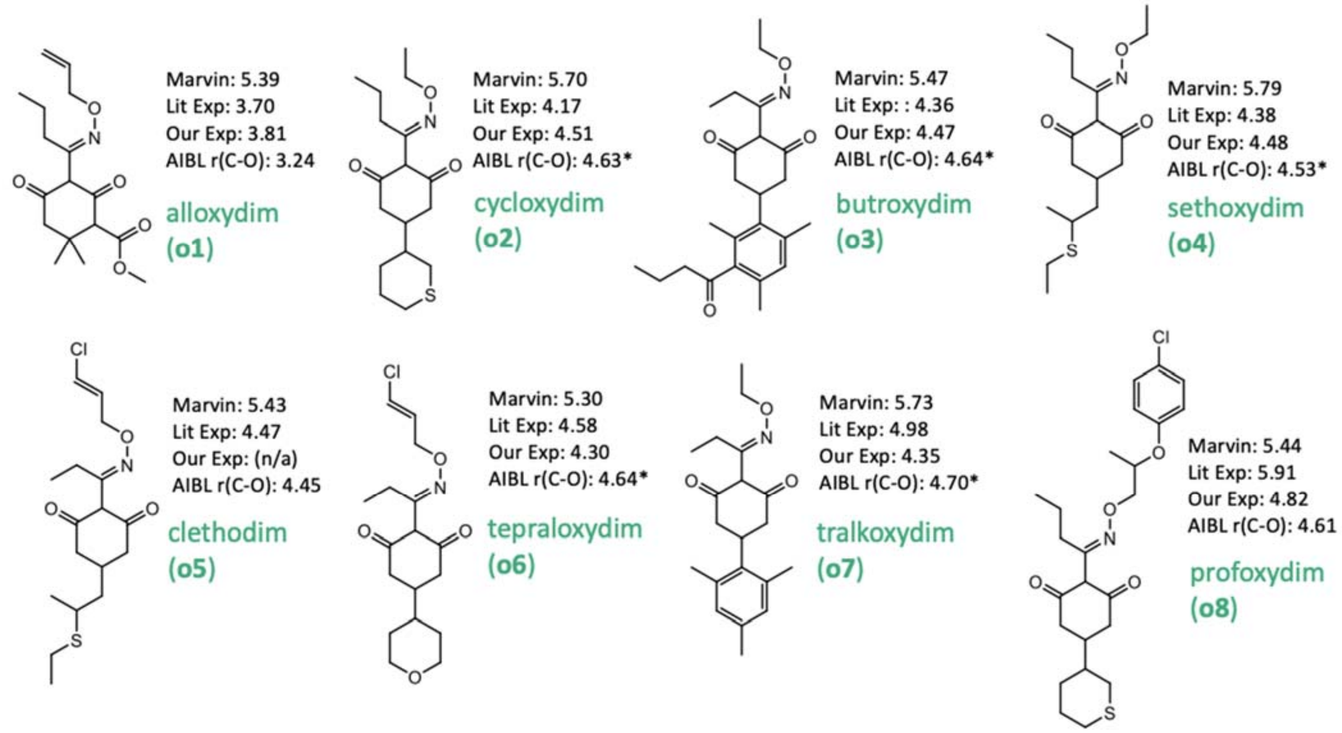

Supplementary Figure 3. For the series **tet1**-**tet6**: Marvin predictions (no consideration of tautomers/resonance), literature experimental values (“Exp”) and AIBL predictions made using the C-O bond model, i.e. pK_a = 98.38*r(C-O)-127.71. An asterisk (*) denotes predictions for compounds that feature in the test set, i.e. are external predictions. Those without asterisks feature in the training set used to formulate the model itself.

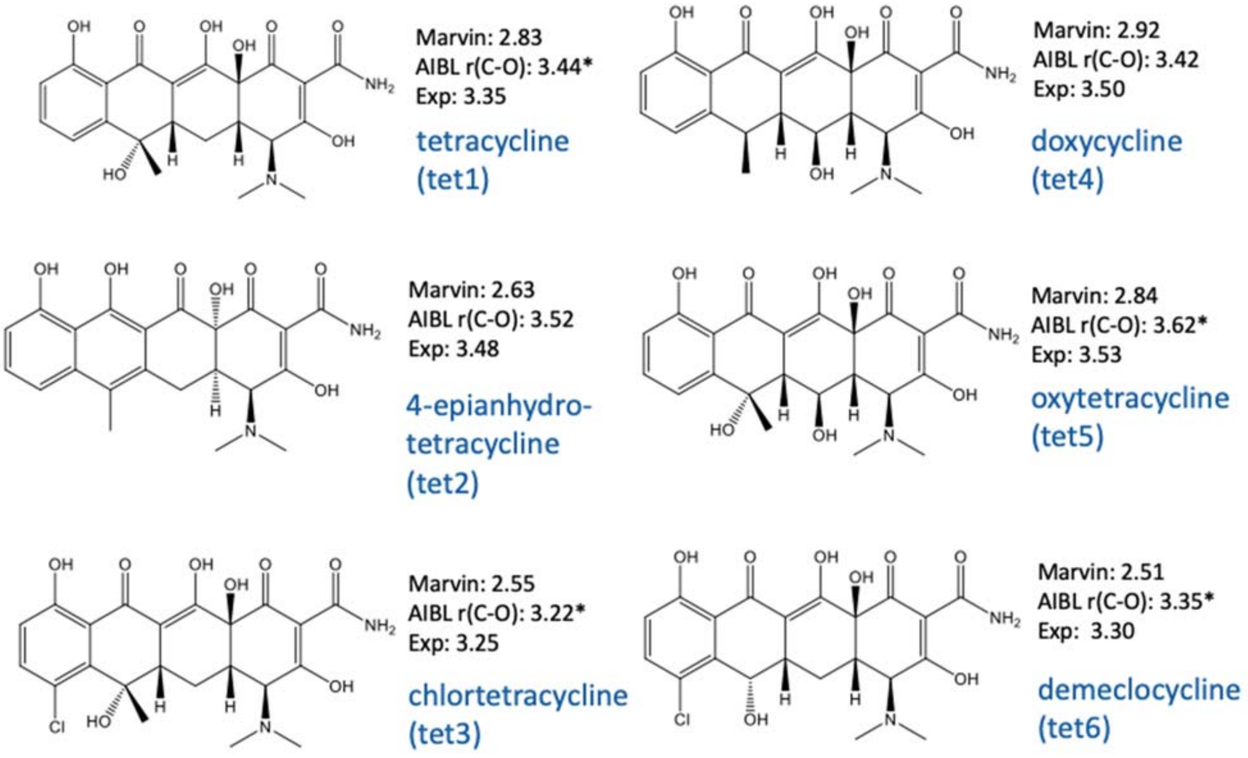

Supplementary References

1. D. L. Lee *et al., Pestic. Sci.*, **1998**, 54, 377-384.
2. Iwataki, I.; Hirono, Y., Synthesis of Pesticides Chemical Structure and Biological Activity Natural Products with Biological Activity: *The Chemical Structure and Herbicidal Activity of Alloxydim-sodium and Related Compounds,* Symposia Papers Presented at the Fourth International Congress of Pesticide Chemistry, Zurich, Switzerland, July 24–28 **1978**, Pages 235-243.
3. Soeda, Y.; Ishihara, K.; Iwataki, I.; Kamimura, H., Fate of a Herbicide 14C-Alloxydim-sodium in Sugar Beet*, J. Pesticide Sci.*, **1978,** 4, 121-128.
4. (No author listed- European Food Safety Authority), Conclusion on the peer review of the pesticide risk assessment of the active substance cycloxydim, EFSA Journal, **2010**, 8, 1669.
5. Tomlin, C., Ed. The Pesticide Manual, 14th ed. Hampshire, UK, British Crop Production Council, **2014**, 134–135.
6. Chamberlain, K.; Evans, A. A.; Bromilow, R. H., 1-Octanol/Water Partition Coefficient (Kow) and pK_a, for Ionisable Pesticides Measured Metric Method, *Pesticide Science*, **1996,** 47, 265-272.
7. Gaillard, J.; Thomas, M.; Lazartigues, A.; Bonnefille, B.; Pallez, C.; Dauchy, X.; Feidt, C.; Banas. D., Potential of barrage fish ponds for the mitigation of pesticide pollution in streams, *Environ. Sci. Pollut. Res.,* **2016,** 23–35.
8. Tomlin CDS., Ed. The Pesticide Manual: A World Compendium. 14 Ed., Hampshire: BCPC Publications; **2006.**
9. Şanli, S.; Şanli, N.; Alsancak, G., Determination of Protonation Constants of Some Tetracycline Antibiotics by Potentiometry and LC Methods in Water and Acetonitrile-Water Binary Mixtures*, J. Braz. Chem. Soc.*, **2009,** 20, 939-946.
10. Benet, L. Z.; Goyan, J. E, Determination of the Stability Constants of Tetracycline Complexes, *J. Pharm. Sci.,* **1965**, 54, 983-987.
11. Roy, K.; Das, N.; Ambure, P.; Aher, R. B., *Chemo. Intell. Lab. Syst.,* **2016**, 152, 18-33.
12. Shedlovsky, T. In *Electrolytes;* Peace, B., Ed.; Pergamon Press, New York, USA, **1962**; pp 146-151.
13. Yasuda, M. *Bull. Chem. Soc. Jpn.* **1959**, 32, 429-432
